# Distance Constraint Ensemble Kalman Filter for Pedestrian Localization

**DOI:** 10.3390/mi17040436

**Published:** 2026-03-31

**Authors:** Lei Deng, Jingwen Yu, Manman Li, Qingao Zhao, Yuan Xu

**Affiliations:** 1 School of Electrical Engineering, Shandong Huayu University of Technology, Dezhou 253034, China; 2 Dezhou Intelligent Equipment Research and Development Center, Dezhou 253034, China; 3 School of Electrical Engineering, University of Jinan, Jinan 250022, China; 4 Jinan Chenhe Information Technology Co., Ltd., Jinan 250022, China

**Keywords:** pedestrian localization, distance constraint, ensemble extended Kalman filter, inertial navigation system

## Abstract

To enhance the positioning accuracy of the inertial measurement unit (IMU)-based pedestrian localization, this study proposes an adaptive ensemble extended Kalman filter (EnEKF) that incorporates a distance constraint (DC). This study first introduces a dual foot-mounted IMU-based pedestrian localization system that employs two IMUs to measure the target human’s position. Second, an augmented data fusion model is developed by incorporating attitude quaternions from the inertial navigation system (INS) into the conventional INS error-state vector. Based on this new data fusion model, a DC-based EnEKF is designed. In this method, the EnEKF employs ensemble factors to address nonlinear and non-Gaussian characteristics inherent in the data fusion process. Then, the colored measurement noise (CMN) is considered, and the method is modified to form an EnEKF under CMN (cEnEKF). Moreover, the DC is employed to further restrict the INS-derived position estimates of the left and right feet obtained from the EnEKF algorithm. Finally, validation in two real-world scenarios confirms the effectiveness and superior performance of the proposed approach.

## 1. Introduction

In recent years, the demand for high-precision personnel localization has surged [1]. For instance, firefighters must obtain precise location information in complex fire environments to develop detailed rescue plans. Hospitals must accurately locate patients in the hospital area to effectively supervise the patients. In [2], self-precise human position information was measured using inertial measurement units (IMUs). Developing methods for utilizing limited sensor data to obtain accurate target pedestrian position information has gradually become a research hotspot in this field [3,4].

Numerous techniques have recently been developed to enhance pedestrian positioning accuracy. From a technological viewpoint, current mainstream solutions include global navigation satellite systems (GNSSs) [5], radiofrequency identification (RFID) [6], ultrawideband (UWB) [7], and WiFi [8]. For example, an extended Kalman filter (EKF) that integrates the maximum correntropy criterion (MCC) with variational Bayesian (VB) approximation was used in a previous study [9] to improve state estimation performance under time-varying GNSS observation conditions. In [10], multi-GNSS integration was explored to improve the state estimation for uncrewed aerial vehicles (UAVs). Wu et al. [11] employed a custom-designed GNSS receiver to assess the accuracy of a common-view time transfer algorithm combining Kalman filtering (KF) with Rauch–Tung–Striebel (RTS) smoothing. They noted that GNSS-based methods can provide stable solutions; however, their limitation is that the signal is affected by the urban canyon effect and closed environments. To address this problem, RFID-, UWB-, and WiFi-based methods have been proposed. For instance, passive RFID tags have been utilized to achieve efficient localization and pose estimation in mobile robot navigation [6,12]. Indoor navigation aids for visually impaired and elderly individuals using RFID technology have been developed [13]. In parallel, UWB systems employing distributed adaptive EFIR filtering have been shown to substantially enhance indoor robot positioning accuracy [14]. A UWB-based localization system for indoor robot navigation was proposed in [15]. In [16], a tightly-coupled UWB–IMU–odometer fusion approach was presented for robust robot localization in mixed line-of-sight (LOS)/non-line-of-sight (NLOS) environments. Furthermore, ref. [17] investigated a method that achieves mobile robot localization using only a single UWB anchor. Notably, the alternative method to GNSS requires the pre-arrangement of reference nodes with known positions, which is not easily achievable in practice. An inertial navigation system (INS) is introduced to maintain high localization accuracy. For example, in [18], a loosely coupled global positioning system (GPS)–INS integrated navigation approach was developed. A long short-term memory (LSTM)–recurrent fuzzy wavelet KF was proposed in [19]. In [20], the integration of the Chinese BeiDou Navigation Satellite System (BDS) and INS was proposed. Although the INS is used to maintain seamless navigation, its self-navigation model is considerably useful in special scenarios. For example, during firefighting operations, firefighters extinguish fires on-site; INS-based methods are useful in such scenarios.

By employing data fusion filters, the positioning accuracy can be further enhanced [21]. KF is a widely used data fusion technique for integrating sensor data in navigation systems. For example, ref. [22] developed an out-of-sequence measurement (OOSM)-adaptive Sequential Tobit KF (OS–TKF) to address the filtering challenges associated with censored and OOSMs within distributed X-ray pulsar-based navigation systems. In [23], a modified adaptive factor-based KF was proposed for the continuous urban precise pointing positioning. However, the aforementioned KF method fails to account for colored measurement noise (CMN). To address this limitation, ref. [24] proposed a modified KF based on the backward Euler (BE) method, which is specifically tailored for models involving CMN and offers a better fit for systems without feedback. Meanwhile, to achieve precise position information in INS-based methodologies, the implementation of a constraint filter has been introduced. For example, ref. [25] proposed a distance constraint (DC)-enhanced dual-foot-mounted INS for pedestrian localization. In this study, this method is improved under CMN [26].

In [26], we have proposed a DC-KF method under CMN. However, it should be pointed out that we used attitude angles to calculate the transition matrix in that paper. Although this method is able to obtain the transition matrix, its robustness is worse. In this work, we employ the quaternions instead of attitude angles to calculate the attitude transition matrix. Moreover, we add the quaternions to the state vector, and the data fusion model becomes a nonlinear model. To achieve high-precision pedestrian localization, this study proposes a DC adaptive ensemble extended KF (EnEKF), as presented in the next section. First, the design of the new data fusion model is introduced. Then, an EnEKF under CMN (cEnEKF), incorporating a switching factor, is developed. Finally, real-world experiments demonstrate the remarkable performance of the proposed algorithm.

- Establishment of the new data fusion model The state vector includes the standard INS errors (position, velocity, accelerometer bias, and gyroscope bias), and the attitude quaternion generated during the INS integration process is appended to the classical INS error-state vector. During the stance phase, the INS-propagated velocity and the quaternions are employed as the measurement of the data fusion model.
- Design of the DC-based EnEKF under CMN (cEnEKF) In this method, the EnEKF employs ensemble factors to address nonlinear and non-Gaussian problems in data fusion models. Then, the CMN is considered, and the method is modified to form an EnEKF under CMN (cEnEKF). Moreover, the DC is employed to further restrict the left- and right-foot positions estimated by the EnEKF algorithm.
- Evaluation of the proposed localization method using two distinct trajectories, where one path is traversed repeatedly in four separate trials.

The rest of this paper is organized as follows. Section 2 reviews the fundamental principles of bipedal inertial navigation using dual foot-mounted IMUs. The proposed cEnEKF is derived in detail in Section 3. Section 4 reports the experimental setup and corresponding performance analysis. Concluding remarks are provided in Section 5.

## 2. Pedestrian Navigation Employing Dual Foot-Mounted IMUs

This section presents the technical framework and system architecture of the proposed dual-foot IMU-based pedestrian localization system. Furthermore, the state and measurement models adopted in the developed filtering algorithm are described in detail.

### 2.1. Dual-Foot Inertial Navigation for Pedestrian Localization

**Figure 1:**
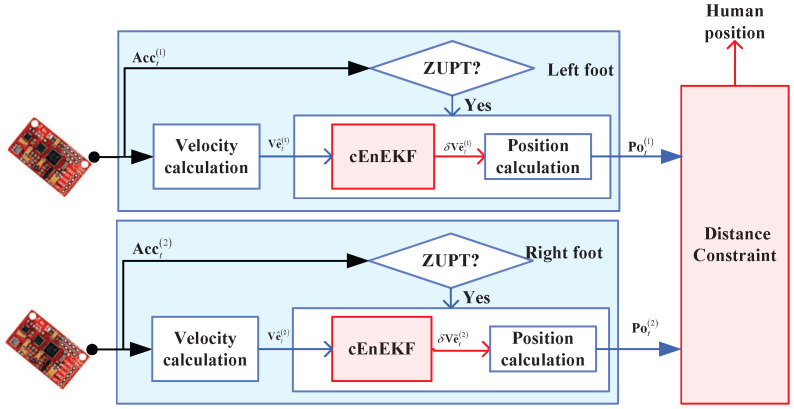
Diagram of the distance constraint (DC) adaptive ensemble extended Kalman filter (EnKF) for pedestrian localization under colored measurement noise (CMN).

This subsection introduces the proposed pedestrian navigation framework utilizing dual foot-mounted IMUs. The flow diagram of the dual foot-mounted IMU-based pedestrian navigation method employing the DC adaptive ensemble EnKF under CMN (cEnEKF) is shown in Figure 1. This study first employs two foot-mounted IMUs. Then, the cEnEKF is employed to correct the velocities ${V\hat{e}}_{k}^{L}$ and ${V\hat{e}}_{k}^{R}$ of the feet derived from the INS solution when the left and right feet are at zero velocity update (ZUPT). Here, *L* and *R* denote the left and right feet, respectively. Moreover, the DC is applied to further reduce the localization error, and the final human position is obtained.

### 2.2. State and Measurement Equations

Building upon the system model presented in Section 2.1, the state and measurement models for the proposed cEnEKF are derived below. The state transition equation adopted for the left foot is expressed by Equation (1).

(1) $$\underset{s_{k}^{L}}{\underset{︸}{\left[\begin{array}{c} q_{k}^{L}\\ \delta P_{k}^{\left(n\right)L}\\ \delta V_{k}^{\left(n\right)L}\\ \delta \nabla_{k}^{\left(b\right)L}\\ \delta \varepsilon_{k}^{\left(b\right)L} \end{array}\right]}}=\underset{f\left(s_{k-1}^{L}\right)}{\underset{︸}{\left[\begin{array}{c} q_{k-1}^{L}+\frac{1}{2}\Omega_{k}\Delta T\\ \delta P_{k-1}^{\left(n\right)L}+\Delta T\delta V_{k-1}^{\left(n\right)}\\ \delta V_{k-1}^{\left(n\right)L}+\Delta TC_{b}^{n}\delta \nabla_{k-1}^{\left(b\right)}\\ \delta \nabla_{k-1}^{\left(b\right)L}\\ \delta \varepsilon_{k-1}^{\left(b\right)L} \end{array}\right]}}+w_{k}^{L}\;,$$

(2) $$\begin{array}{c} C_{b}^{n}=\left[\begin{array}{c} {\left(q_{0,k}^{L}\right)}^{2}+{\left(q_{1,k}^{L}\right)}^{2}-{\left(q_{2,k}^{L}\right)}^{2}-{\left(q_{3,k}^{L}\right)}^{2}\\ 2\left(q_{1,k}^{L}q_{2,k}^{L}-q_{0,k}^{L}q_{3,k}^{L}\right)\\ 2\left(q_{1,k}^{L}q_{3,k}^{L}+q_{0,k}^{L}q_{2,k}^{L}\right) \end{array}\right.\\ \left.\begin{array}{cc} 2\left(q_{1,k}^{L}q_{2,k}^{L}+q_{0,k}^{L}q_{3,k}^{L}\right) & 2\left(q_{1,k}^{L}q_{3,k}^{L}-q_{0,k}^{L}q_{2,k}^{L}\right)\\ {\left(q_{0,k}^{L}\right)}^{2}-{\left(q_{1,k}^{L}\right)}^{2}+{\left(q_{2,k}^{L}\right)}^{2}-{\left(q_{3,k}^{L}\right)}^{2} & 2\left(q_{2,k}^{L}q_{3,k}^{L}+q_{0,k}^{L}q_{1,k}^{L}\right)\\ 2\left(q_{2,k}^{L}q_{3,k}^{L}-q_{0,k}^{L}q_{1,k}^{L}\right) & {\left(q_{0,k}^{L}\right)}^{2}-{\left(q_{1,k}^{L}\right)}^{2}-{\left(q_{2,k}^{L}\right)}^{2}+{\left(q_{3,k}^{L}\right)}^{2} \end{array}\right] \end{array}\;,$$

where $C_{b}^{n}$ is the rotation matrix, *b* means the body-frame (b-frame), *n* means the east-north-up navigation-frame (n-frame), $q_{k}^{L}={\left[\begin{array}{cccc} q_{0,k}^{L} & q_{1,k}^{L} & q_{2,k}^{L} & q_{3,k}^{L} \end{array}\right]}^{T}$ is the quaternion of the left foot at the time index *k*, $\Omega_{k}=\left[\begin{array}{cccc} 0 & -\omega_{x} & -\omega_{y} & -\omega_{z}\\ \omega_{x} & 0 & \omega_{z} & -\omega_{y}\\ \omega_{y} & -\omega_{z} & 0 & \omega_{x}\\ \omega_{z} & \omega_{y} & -\omega_{x} & 0 \end{array}\right]$ is the quaternion derivative matrix, $\left[\begin{array}{ccc} \omega_{x} & \omega_{y} & \omega_{z} \end{array}\right]$ is the angular velocity of the gyroscope in three directions, ΔT is the sampling time, $\delta P_{k}^{\left(n\right)L}$ is the left foot’s position error derived by the INS in navigation-frame (n-frame) at the time index *k*, $\delta V_{k}^{\left(n\right)L}$ is the left foot’s velocity error derived by the INS in navigation-frame (n-frame) at the time index *k*, $\delta \nabla_{k}^{\left(b\right)L}$ stands for the accelerometer bias expressed in the body frame (b-frame) at the time index *k*, $\delta \varepsilon_{k}^{\left(b\right)L}$ denotes the gyroscope bias in the body frame (b-frame) at the time index *k*, and $w_{k}^{L}∼N\left(0,Q_{k}^{L}\right)$ is the *Gaussian* system noise with the system covariance $Q_{k}^{L}$.

Here, the sum of the values along the three axes in the b-frame is used as the initial reference for ZUPT, calculated using the following equations:

(3) $$\begin{array}{c} Z_{th\_down}<∥f_{x}^{L}+f_{y}^{L}+f_{z}^{L}∥<Z_{th\_up}\;, \end{array}$$

where $f_{k}^{L}=\left[\begin{array}{ccc} f_{x}^{L} & f_{y}^{L} & f_{z}^{L} \end{array}\right]$ is the accelerometer under the b-frame for the left foot, and $\left(Z_{th\_down},Z_{th\_up}\right)$ is the threshold for the ZUPT. When ZUPT is enabled, the measurement equation is derived as follows:

(4) $$\underset{y_{k}^{L}}{\underset{︸}{\left[\begin{array}{c} \delta V_{k}^{\left(n\right)L}\\ q_{k}^{L} \end{array}\right]}}=\left[\begin{array}{c} V_{k}^{\left(n\right)L}-0\\ q_{k}^{L} \end{array}\right]=\underset{H^{L}}{\underset{︸}{\left[\begin{array}{ccccc} 0_{3\times 4} & I_{3\times 3} & 0_{3\times 3} & 0_{3\times 3} & 0_{3\times 3}\\ I_{4\times 4} & 0_{4\times 3} & 0_{4\times 3} & 0_{4\times 3} & 0_{4\times 3} \end{array}\right]}}s_{k}^{L}+\underset{V_{k}^{L}}{\underset{︸}{\phi V_{k-1}^{L}+\delta_{k}^{L}}}\;,$$

where $V_{k}^{L}$ is the CMN for the left foot at the left foot, ϕ is the colored factor, and $\delta_{k}^{L}∼N\left(0,R_{k}^{L}\right)$ is the *Gaussian* noise with the measurement covariance $R_{k}^{L}$.

## 3. Ensemble Extended Kalman Filter Under CMN

Building upon Equations (1) and (4), state augmentation is introduced to establish an augmented framework that can withstand colored measurement noise. Subsequently, an ensemble extended Kalman filter based on the new data fusion model is developed.

### 3.1. State Augmentation

In this subsection, the new data fusion model is derived. From Equations (1) and (4), we can obtain the new state and measurement equations (Equations (5) and (6)) in an augmented state.

(5) $$\underset{{\bar{s}}_{k}^{L}}{\underset{︸}{\left[\begin{array}{c} s_{k}^{L}\\ V_{k}^{L} \end{array}\right]}}=\underset{\bar{f}\left({\bar{s}}_{k-1}^{L}\right)}{\underset{︸}{\left[\begin{array}{c} f\left(s_{k-1}^{L}\right)\\ \phi V_{k-1}^{L} \end{array}\right]}}+\underset{{\bar{w}}_{k}^{L}}{\underset{︸}{\left[\begin{array}{c} w_{k}^{L}\\ \delta_{k}^{L} \end{array}\right]}}\;,$$

(6) $${\bar{y}}_{k}^{L}=\left[\begin{array}{cc} H^{L} & I \end{array}\right]{\bar{s}}_{k}^{L}+0={\bar{H}}^{L}{\bar{s}}_{k}^{L}+{\bar{V}}_{k}^{L}\;,$$

where ${\bar{V}}_{k}^{L}=0$, and its covariance is $E\left\{{\bar{V}}_{k}^{L}{\left({\bar{V}}_{k}^{L}\right)}^{T}\right\}=0$, and the covariance of the ${\bar{w}}_{k}^{L}$ can be computed as follows:

(7) $${\bar{Q}}_{k}^{L}=E\left\{{\bar{w}}_{k}^{L}{\left({\bar{w}}_{k}^{L}\right)}^{T}\right\}=\left[\begin{array}{cc} Q_{k}^{L} & 0\\ 0 & R_{k}^{L} \end{array}\right]\;,$$

### 3.2. Ensemble Extended Kalman Filter

Based on Equations (5) and (6), the ensemble extended Kalman filter (EnEKF) is designed in the following section. The EnEKF is a filtering technology for nonlinear system state estimation, especially for high-dimensional systems and large-scale data processing [27]. Moreover, it approximates the posterior probability distribution of the system state through a set of samples called “sets” ${\bar{s}}_{k}^{Lj},j\in \left[1,M\right]$, where *M* is the number of “sets”. This approximation includes two core steps:

The first step is the prediction. To determine *M* “sets”, we derive the following:

(8) $${\bar{s}}_{k}^{fj,L}=\bar{f}\left({\bar{s}}_{k-1}^{Lj}\right)+q_{k}^{Lj},q_{k}^{Lj}∼N\left(0,Q_{k}^{L}\right)\;,j\in \left[1,M\right]\;,$$

Meanwhile, we can compute the mean of the state prediction:

(9) $${\bar{s}}_{k}^{f,L}=\frac{1}{M}\sum_{j=1}^{M}{\bar{s}}_{k}^{f,Lj}\;,$$

Then, we form an observation for the state prediction of *M* samples:

(10) $${\bar{y}}_{k}^{f,Lj}={\bar{H}}^{L}{\bar{s}}_{k}^{f,Lj}\;,j\in \left[1,M\right]$$

(11) $${\bar{y}}_{k}^{f,L}=\frac{1}{M}\sum_{j=1}^{M}{\bar{y}}_{k}^{f,Lj}\;,$$

The error matrix of the state and observation prediction sets can be computed as follows:

(12) $$SE_{k}^{f,L}=\left[\begin{array}{cccc} {\bar{s}}_{k}^{f1,L}-{\bar{s}}_{k}^{f,L} & {\bar{s}}_{k}^{f2,L}-{\bar{s}}_{k}^{f,L} & \cdots & {\bar{s}}_{k}^{fM,L}-{\bar{s}}_{k}^{f,L} \end{array}\right]\;,$$

(13) $$SY_{k}^{f,L}=\left[\begin{array}{cccc} {\bar{y}}_{k}^{f1,L}-{\bar{y}}_{k}^{f,L} & {\bar{y}}_{k}^{f2,L}-{\bar{y}}_{k}^{f,L} & \cdots & {\bar{y}}_{k}^{fM,L}-{\bar{y}}_{k}^{f,L} \end{array}\right]\;,$$

The second step is the analysis. First, we calculate the error covariance:

(14) $$P_{SESY,k}^{f,L}=\frac{1}{M-1}{\mathrm{SE}}_{k}^{f,L}{\left({\mathrm{SY}}_{k}^{f,L}\right)}^{T}\;,$$

(15) $$P_{SYSY,k}^{f,L}=\frac{1}{M-1}{\mathrm{SY}}_{k}^{f,L}{\left({\mathrm{SY}}_{k}^{f,L}\right)}^{T}\;,$$

The filter gain is given by the expression below:

(16) $$K_{k}^{L}=P_{SESY,k}^{f,L}{\left(P_{SYSY,k}^{f,L}+{\bar{R}}_{k}^{L}\right)}^{-1}\;,$$

where ${\bar{R}}_{k}^{L}$ is the covariance. The sample state prediction can be computed using the sample state estimation.

(17) $${\bar{s}}_{k}^{aj,L}={\bar{s}}_{k}^{fj,L}+K_{k}^{L}\left({\bar{y}}_{k}^{L}-{\bar{y}}_{k}^{Lj}\right)\;,j\in \left[1,M\right]\;,$$

Finally, the output of the EnEKF can be obtained through the following expression:

(18) $${\bar{s}}_{k}^{a,L}=\frac{1}{M}\sum_{j=1}^{M}{\bar{s}}_{k}^{aj,L}\;,j\in \left[1,M\right]\;,$$

Here, we can obtain the left foot’s position estimation. The cEnEKF for the left foot is presented as Algorithm 1. If we replace ‘L’ with ‘R’ in the equations mentioned above, we can easily obtain the right foot’s position.
**Algorithm 1:** Left-foot cEnEKF
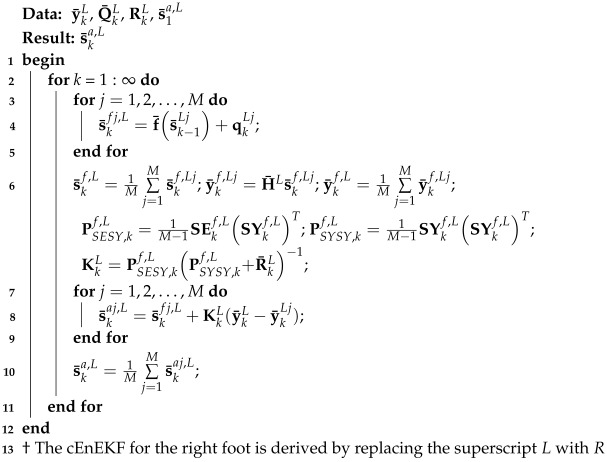


### 3.3. Distance-Constraint cEnEKF

As mentioned in the previous subsection, the results demonstrate that the proposed cEnEKF can improve the positioning accuracy for foot-mounted IMU-based pedestrian localization. Investigating methods to introduce additional constraints to the INS is an important research hotspot in this field. In this study, the distance between feet is considered to add physical constraints to the cEnEKF. If the left and right feet positions are obtained, the distance error $\delta d_{ff}$ between the left and right feet can be computed.

(19) $$\begin{array}{c} d_{ff}=\left[\begin{array}{c} \delta Po_{x,k}^{L}-\delta Po_{x,k}^{R}\\ \delta Po_{y,k}^{L}-\delta Po_{y,k}^{R} \end{array}\right] \end{array}\;$$

where $\left(Po_{x,k}^{L},Po_{y,k}^{L}\right)$ and $\left(Po_{x,k}^{R},Po_{y,k}^{R}\right)$ represent the east and north positions, respectively, measured by the IMUs mounted on the left foot and right feet. Here, we set ${\mathrm{Po}}_{k}^{ff}={\left[\begin{array}{cc} {\mathrm{Po}}_{k}^{L} & {\mathrm{Po}}_{k}^{R} \end{array}\right]}^{T}$, The state estimation for the dual-foot localization system, subject to inter-foot distance constraints, is formulated as the following optimization problem:

(20) $${\mathrm{ffi}\bar{P}o}_{k}^{ff}=\underset{{\mathrm{ffi}\overset{⌢}{P}o}_{k}^{ff}}{\arg\min}{\left({\mathrm{ffi}\overset{⌢}{P}o}_{k}^{ff}-{\mathrm{ffiPo}}_{k}^{ff}\right)}^{T}{P_{k}^{ff}}^{-1}\left({\mathrm{ffi}\overset{⌢}{P}o}_{k}^{ff}-{\mathrm{ffiPo}}_{k}^{ff}\right),$$

(21) $$\begin{array}{c} s.t.\;D_{k}{\mathrm{ffiPo}}_{k}^{ff}=d_{k} \end{array}\;,$$

where $P_{k}^{ff}=\left[\begin{array}{cc} P_{k}^{L} & 0\\ 0 & P_{k}^{R} \end{array}\right]$, ${\mathrm{ffiPo}}_{k}^{ff}$’s constrained estimate is denoted as ${\mathrm{ffi}\bar{P}o}_{k}^{ff}$, ${\mathrm{ffi}\overset{⌢}{P}o}_{k}^{ff}$ is the position error computed by the INS and the filter’s output, and $D_{k}[\begin{array}{ccccc} 0_{2\times 7} & I_{2\times 2} & 0_{3\times 14} & -I_{2\times 2} & 0_{3\times 7} \end{array}]$, $d_{k}{\left[\begin{array}{cc} \delta Po_{x,k}^{L}-\delta Po_{x,k}^{R} & \delta Po_{y,k}^{L}-\delta Po_{y,k}^{R} \end{array}\right]}^{T}$. Thus, the solution of problems (20) and (21) can be computed using the following equation:

(22) $$\begin{array}{c} {{\mathrm{ffi}\bar{P}o}_{k}^{ff}=\mathrm{ffi}\overset{⌢}{\;P}o}_{k}^{ff}-P_{k}^{ff}D_{k}^{T}{\left[D_{k}P_{k}^{ff}D_{k}^{T}\right]}^{-1}\left({D_{k}\mathrm{ffi}\overset{⌢}{\;P}o}_{k}^{ff}-d_{k}\right), \end{array}$$

## 4. Test

This section presents a real-time experiment that evaluates the effectiveness of the proposed approach. The experimental setup is first presented, followed by an analysis of the effectiveness of the proposed approach.

### 4.1. Experimental Setup

**Figure 2:**
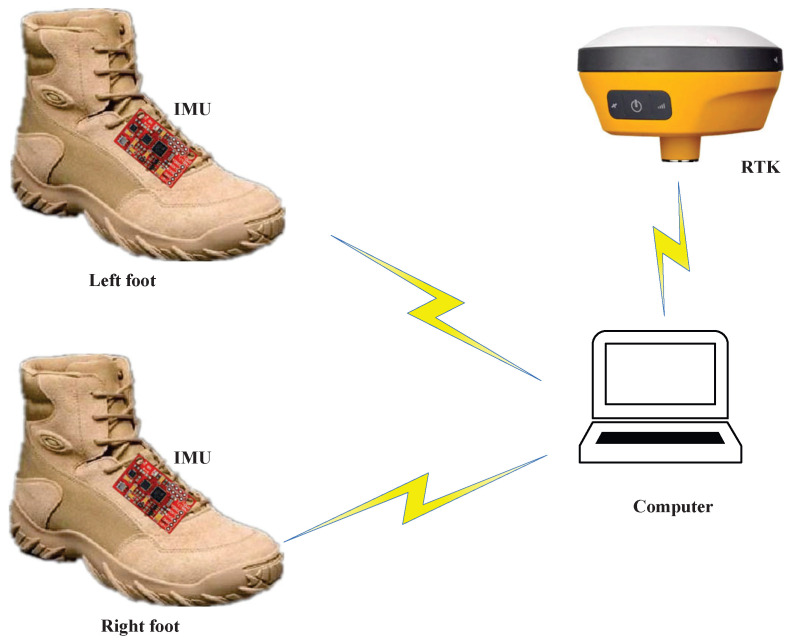
Diagram of the testbed used in the study.

**Figure 3:**
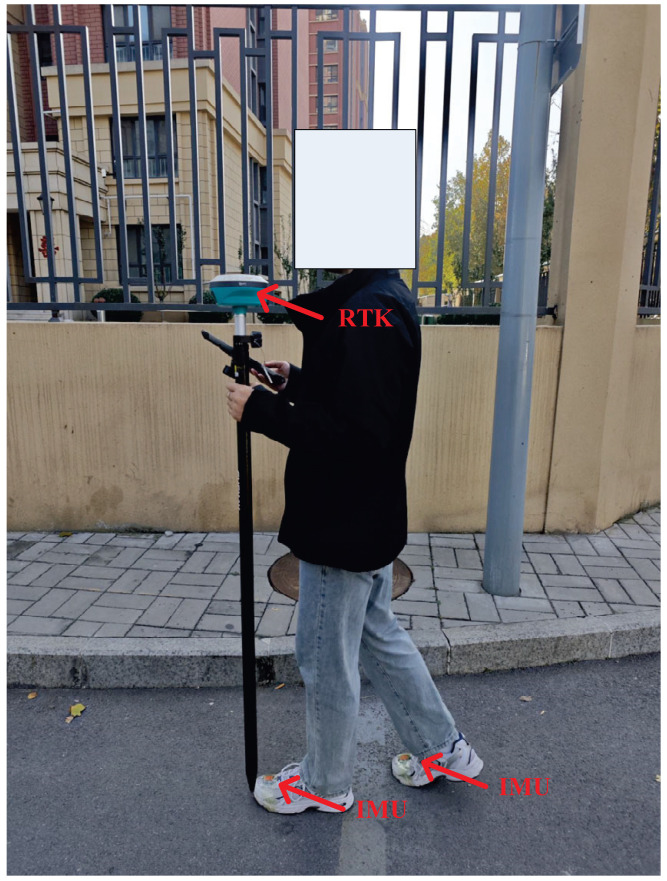
Physical image of the experimental platform.

In this subsection, the test setup is presented. The same testbed that was used in [26] was employed. The block diagram of the experimental setup used in this article is shown in Figure 2. Physical image of the experimental platform is shown in Figure 3. Furthermore, one IMU was attached to each foot. One R-T-K was used as the reference system to provide reference values. During the tests, when the target human walked following the preset path, all the IMUs measured the target human’s position. Meanwhile, the R-T-K provided the reference value. A computer was used to collect the sensors’ data. The specifications of the IMUs and RTK system adopted in this work are summarized in Table 1 and Table 2. These are the same as those of the sensors used in [26].

### 4.2. Real Test Using Path 1

**Figure 4:**
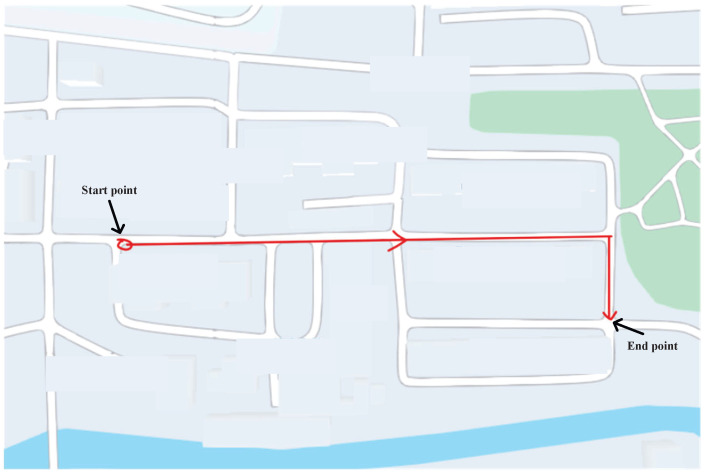
Schematic diagram of Trajectory 1.

**Figure 5:**
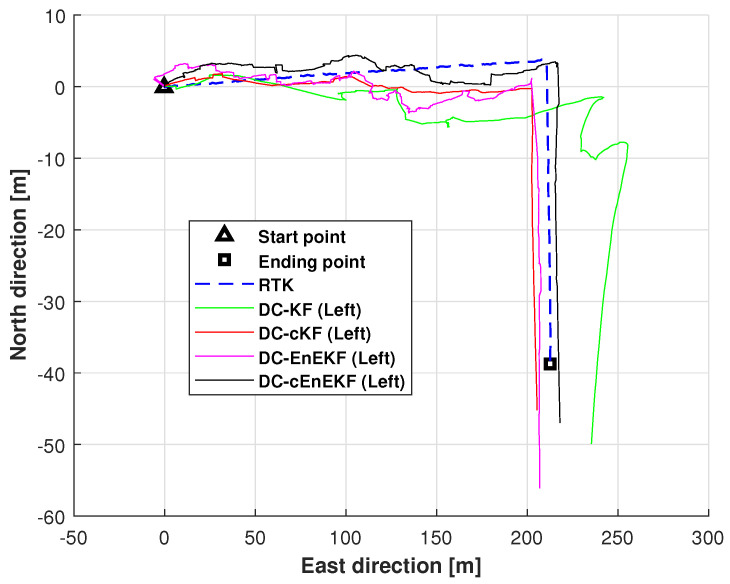
Left-foot trajectories estimated by DC–KF, DC–cKF, DC–EnEKF, and DC–cEnEKF on Path 1.

This subsection evaluates the effectiveness of the proposed approach on Path 1 (Figure 4). Test 1 is performed at the University of Jinan, and the total trajectory length is approximately 240 m, with a sampling time of 0.02 s. Furthermore, the distance constraint Kalman filter (DC–KF), DC–KF under CMN (DC–cKF), and DC–EnEKF are adopted as the benchmarks of the proposed DC-EnEKF under CMN (cEnEKF). The left-foot trajectories obtained with the DC-KF, DC–cKF, DC–EnEKF, and DC–cEnEKF for Path 1 are presented in Figure 5. As shown in the figure, DC–KF exhibits a significant deviation in its estimated trajectory. Compared to DC–KF, the DC–cKF achieves markedly higher accuracy, with its estimated path aligning much more closely with the reference trajectory. The DC–EnEKF has some errors at the initial stage of the trajectory, but its solution quickly approaches the reference path. When compared with the other methods, the proposed DC–cEnEKF’s path is closest to the reference path. The settings of the filter are listed as follows:

(23) $$\begin{array}{ccc} \left[\begin{array}{cc} {\bar{Q}}_{k}^{L} & 0\\ 0 & {\bar{Q}}_{k}^{R} \end{array}\right] & = & \mathrm{diag}\left(\begin{array}{cccc} 0.001\times I_{4\times 4} & 0.75\times I_{3\times 3} & 0.05\times I_{3\times 3} & 0.001\times I_{6\times 6} \end{array}\right.\\ & & \begin{array}{cccc} 0.13\times I_{4\times 4} & 9\times I_{3\times 3} & 0.05\times I_{3\times 3} & 0.01\times I_{6\times 6} \end{array}\\ & & \left.\begin{array}{cccc} 0.09\times I_{3\times 3} & 0.001\times I_{4\times 4} & 0.01\times I_{3\times 3} & 0.0001\times I_{4\times 4} \end{array}\right)\;, \end{array}$$

**Figure 6:**
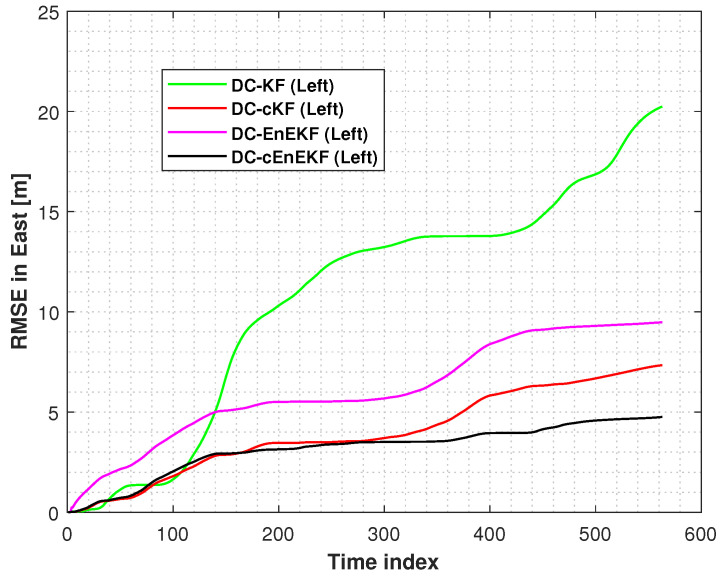
Left-foot eastward position RMSEs obtained with DC–KF, DC–cKF, DC–EnEKF, and DC–cEnEKF on Path 1.

**Figure 7:**
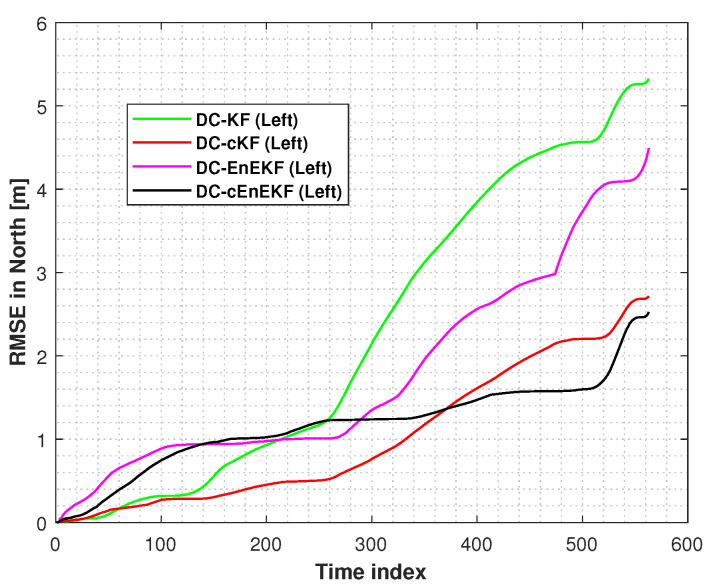
Left-foot northward position RMSEs obtained with the DC–KF, DC–cKF, DC–EnEKF, and DC–cEnEKF on Path 1.

Moreover, the root mean square errors (RMSEs) of the left-foot positioning in the east and north directions, obtained with the DC–KF, DC–cKF, DC–EnEKF, and DC–cEnEKF along Path 1, are presented in Figure 6 and Figure 7, respectively. Figure 6 shows that DC–KF is the largest RME, whereas DC–cKF improves the localization accuracy by considering CMN. Compared with the DC–KF, the DC–EnEKF considerably reduces the localization error, demonstrating that the EnEKF model is effective in improving the localization accuracy. Compared with the EnEKF, the cEnEKF method effectively maintains the positioning accuracy under CMN. However, the EnEKF exhibits the best performance at the beginning of the test, and when exposed to CMN, its localization error improves rapidly. This demonstrates that the proposed method has the best performance.

Figure 7, shows that the conclusions are similar to those made from the results illustrated in Figure 6. The DC–KF has the largest RMEs, and the DC–cKF improves the localization accuracy by considering CMN. Compared with the DC-KF, the DC-EnEKF reduces the localization error considerably, which shows that the EnEKF model is useful for improving the localization accuracy. Compared with the EnEKF, the cEnEKF effectively maintains the positioning accuracy under CMN. However, the EnEKF exhibits the best performance at the beginning of the test, and then, when this method faces CMN, its localization error increases rapidly. Thus, the proposed method exhibits the best performance.

**Figure 8:**
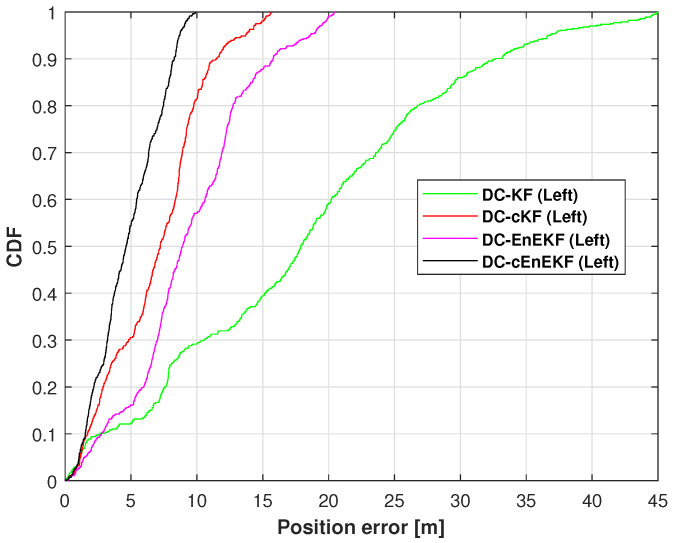
Left-foot position error cumulative distribution functions (CDFs) derived with the DC–KF, DC–cKF, DC–EnEKF, and DC–cEnEKF on Path 1.

The cumulative distribution functions (CDFs) of the left-foot positioning error on Path 1, obtained with the DC–KF, DC–cKF, DC–EnEKF, and DC–cEnEKF, are presented in Figure 8. The superiority of the proposed DC–cEnEKF is quantitatively demonstrated in the figure, where it achieves the smallest localization error at the 0.9 probability mark. In contrast, DC–KF exhibits the largest RME, while the DC–cKF and DC–EnEKF show comparable, intermediate performance. The RMSE values of the left-foot positioning error along Path 1 are summarized in Table 3. According to the table, the proposed DC–cEnEKF achieves a minimum RMSE of 3.64 m. Moreover, it reduces the positioning error by approximately 71.71% compared with DC-KF, and by approximately 47.93% compared with the DC–EnEKF.

### 4.3. Real Test Using Path 2

**Figure 9:**
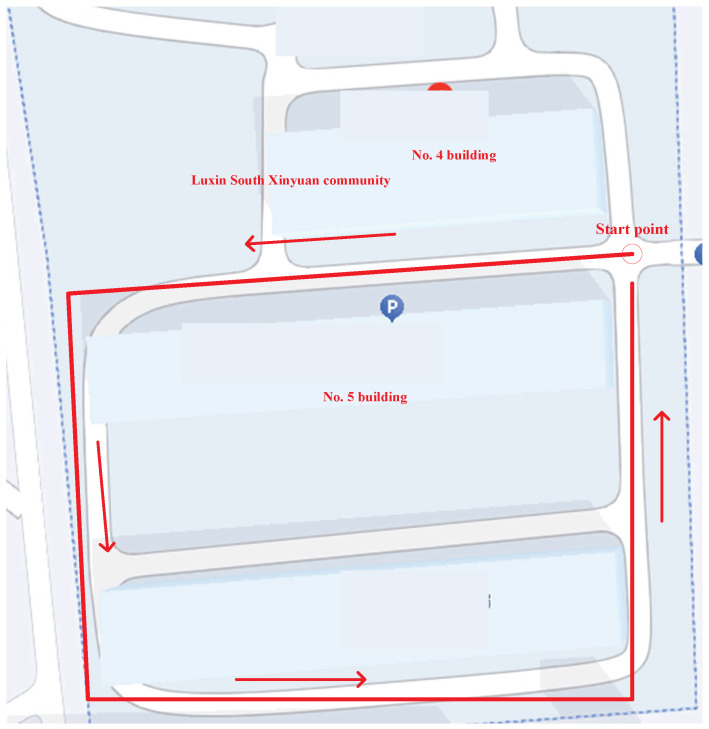
Schematic diagram of Trajectory 2.

In this subsection, the test is repeated following the path shown in Figure 9. The settings of the filter are listed as follows:

(24) $$\begin{array}{ccc} \left[\begin{array}{cc} {\bar{Q}}_{k}^{L} & 0\\ 0 & {\bar{Q}}_{k}^{R} \end{array}\right] & = & \mathrm{diag}\left(\begin{array}{cccc} 0.1\times I_{4\times 4} & 13\times I_{3\times 3} & 0.05\times I_{3\times 3} & 10\times I_{6\times 6} \end{array}\right.\\ & & \begin{array}{cccc} 0.25\times I_{4\times 4} & 5.8\times I_{3\times 3} & 0.05\times I_{3\times 3} & 0.73\times I_{6\times 6} \end{array}\\ & & \left.\begin{array}{cccc} 0.09\times I_{3\times 3} & 0.001\times I_{4\times 4} & 0.01\times I_{3\times 3} & 0.0001\times I_{4\times 4} \end{array}\right)\;, \end{array}$$

**Figure 10:**
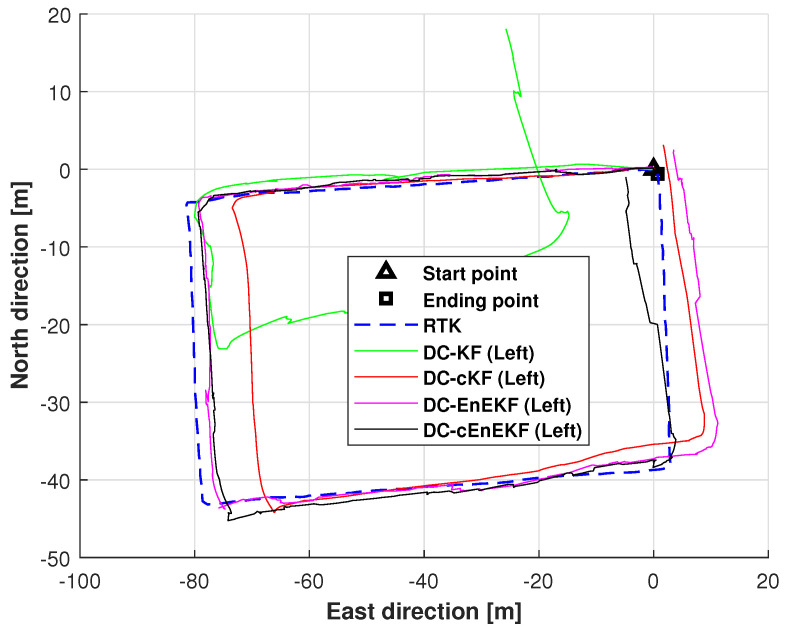
Left-foot trajectories estimated by DC–KF, DC–cKF, DC–EnEKF, and DC–cEnEKF on Path 2.

Figure 10 illustrates the left-foot trajectories estimated by the DC–KF, DC–cKF, DC–EnEKF, and DC–cEnEKF on Path 2. As shown in the figure, both DC–KF and DC–cKF deviate significantly from the reference trajectory, with the DC–KF exhibiting the largest deviation. In contrast, the trajectories produced by the DC–EnEKF and the proposed DC–cEnEKF remain much closer to the ground truth. The proposed DC–cEnEKF demonstrates the highest accuracy, particularly toward the end of Path 2.

**Figure 11:**
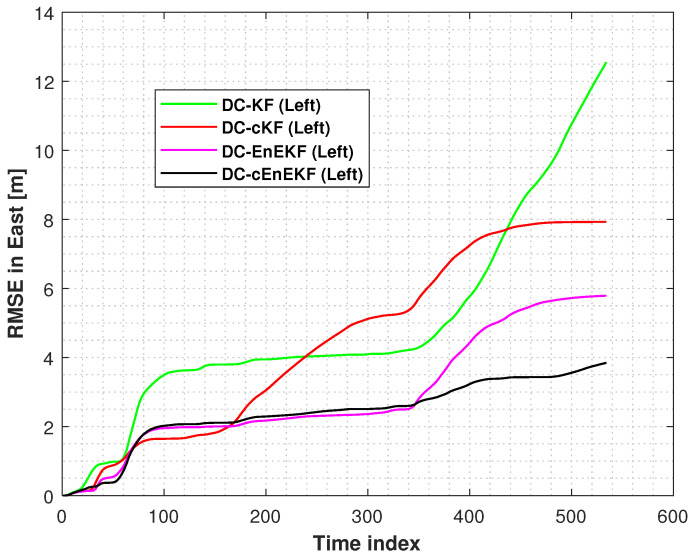
Left-foot eastward position RMSEs obtained with DC–KF, DC–cKF, DC–EnEKF, and DC–cEnEKF on Path 2.

**Figure 12:**
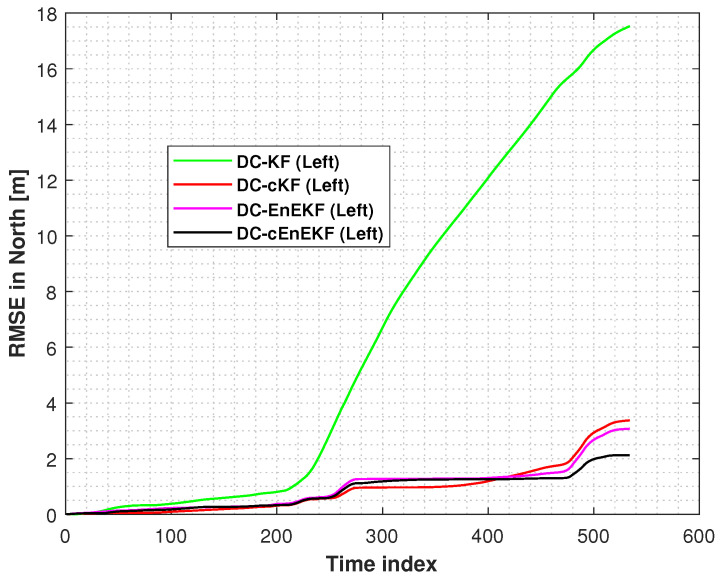
Left-foot northward position RMSEs obtained with DC–KF, DC–cKF, DC–EnEKF, and DC–cEnEKF on Path 2.

Figure 11 and Figure 12 present the RMSEs of the left-foot positioning error in the east and north directions on Path 2, respectively, obtained with the DC–KF, DC–cKF, DC–EnEKF, and DC–cEnEKF. Figure 11 shows that the DC–KF still exhibits the highest error levels. Compared with DC–KF, DC–cKF substantially suppresses the localization error by effectively mitigating the impact of CMN. Both the DC–EnEKF and DC–cEnEKF can reduce the localization error, which shows that the ensemble method can improve the positioning accuracy. Compared with the EnEKF, the cEnEKF method effectively maintains the positioning accuracy under CMN, demonstrating the best overall performance. A similar performance is observed in Figure 12, where the DC–KF exhibits the worst performance, the DC–cKF and DC–EnEKF exhibit comparable performance levels, and the DC–EnEKF exhibits the best performance. Consistent with the results in the east direction, the proposed DC–cEnEKF also achieves the lowest RMSEs in the north direction, further confirming its superior accuracy.

**Figure 13:**
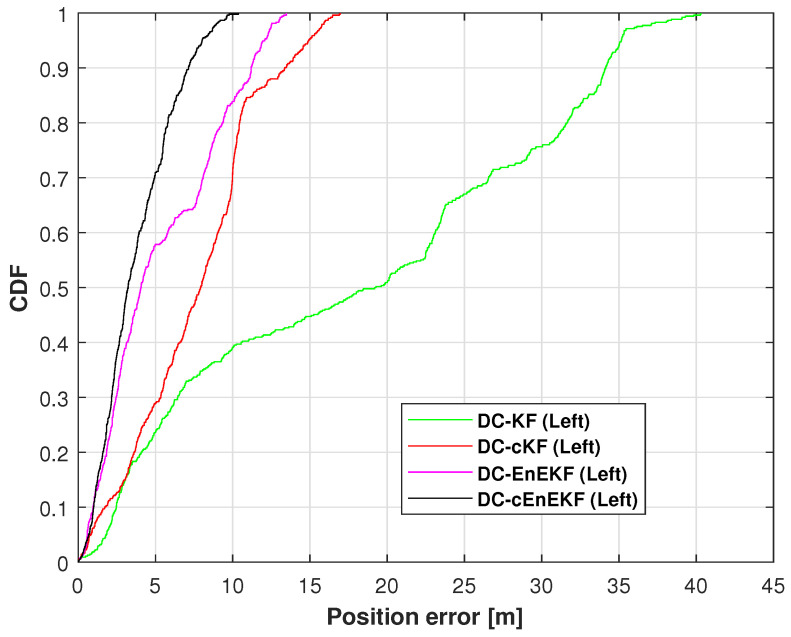
Left-foot position error CDFs derived with DC–KF, DC–cKF, DC–EnEKF, and DC–cEnEKF on Path 2.

Figure 13 displays the CDFs of the left-foot positioning error on Path 2, obtained with the DC–KF, DC–cKF, DC–EnEKF, and DC–cEnEKF. From this figure, The superiority of the proposed DC–cEnEKF is quantitatively demonstrated in the figure, where it achieves the smallest localization error at the 0.9 probability mark. In contrast, DC–KF exhibits the largest error, while the DC–cKF and DC–EnEKF show comparable, intermediate performance. Table 4 lists the RMSE values of the left-foot positioning error on Path 2 for the four algorithms. The results reveal that the proposed DC–cEnEKF attains the minimum RMSE of 2.99 m. In addition, it reduces the positioning error by approximately 80.12% when compared with DC–KF, and by approximately 32.66% compared with DC-EnEKF.

### 4.4. Real Test Using Path 3

**Figure 14:**
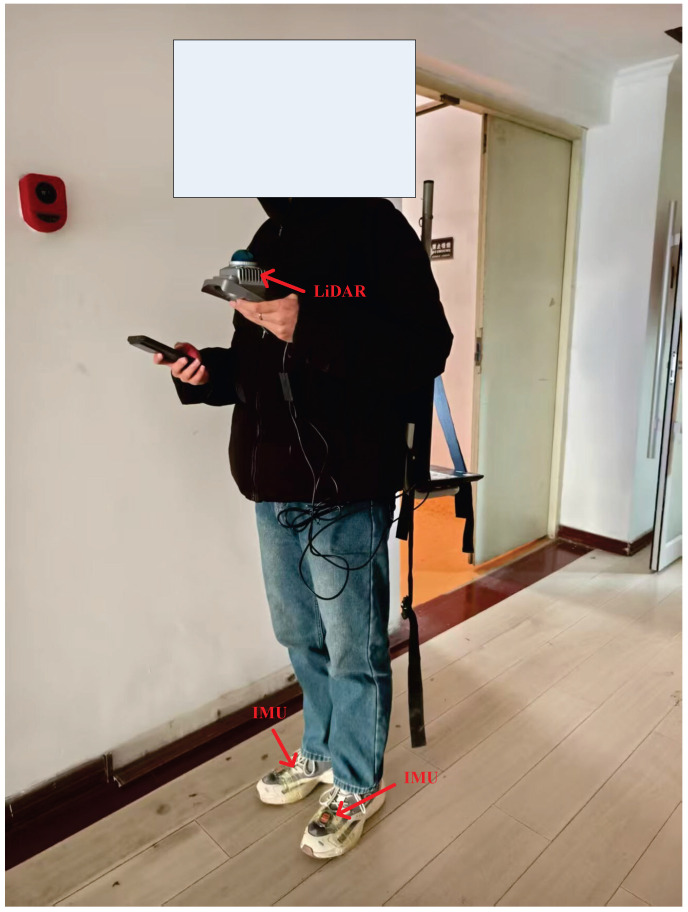
Physical image of the experimental platform for path 3.

**Figure 15:**
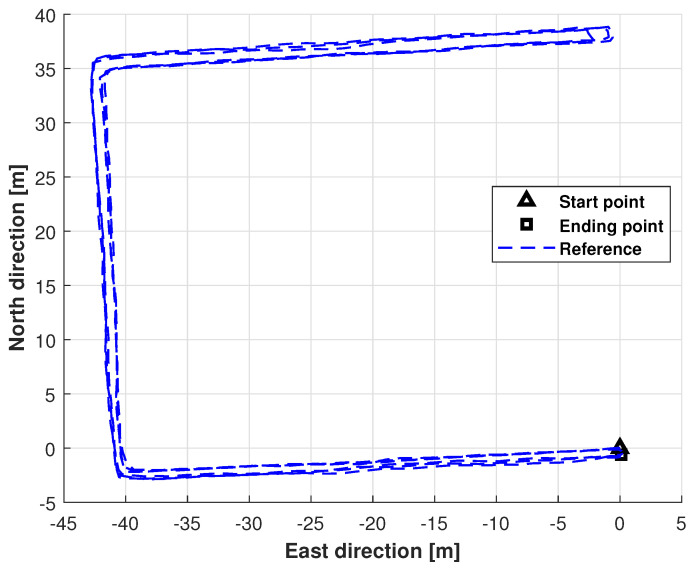
Schematic diagram of Trajectory 3.

In this section, we will investigate the performance of the proposed method via the repeated trajectories in an indoor environment. Figure 14 shows the physical image of the experimental platform for Path 3. Different from tests 1 and 2, in this test, we employ the light detection and ranging (LiDAR) to provide the reference value since the RTK can not obtain the signal in an indoor environment. The Trajectory 3 is shown in Figure 15. In this work, we conducted an indoor test on the fourth floor of Quanjing Tongrun Business Building in Jinan, Shandong Province.

The settings of the filter are listed as follows:

(25) $$\begin{array}{ccc} \left[\begin{array}{cc} {\bar{Q}}_{k}^{L} & 0\\ 0 & {\bar{Q}}_{k}^{R} \end{array}\right] & = & \mathrm{diag}\left(\begin{array}{cccc} 0.07\times I_{4\times 4} & 0.01\times I_{3\times 3} & 0.05\times I_{3\times 3} & 55\times I_{6\times 6} \end{array}\right.\\ & & \begin{array}{cccc} 5\times I_{4\times 4} & I_{3\times 3} & 0.037\times I_{3\times 3} & 65\times I_{6\times 6} \end{array}\\ & & \left.\begin{array}{cccc} 0.09\times I_{3\times 3} & 0.001\times I_{4\times 4} & 0.01\times I_{3\times 3} & 0.0001\times I_{4\times 4} \end{array}\right) \end{array}$$

**Figure 16:**
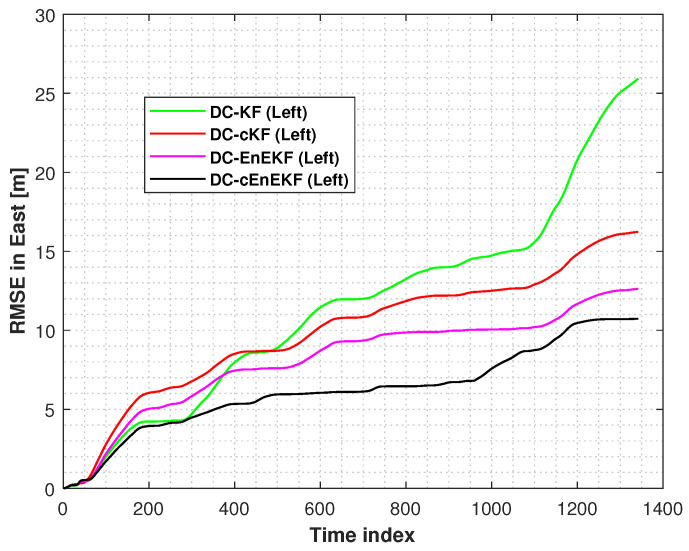
Left-foot eastward position RMSEs obtained with DC–KF, DC–cKF, DC–EnEKF, and DC–cEnEKF on Path 3.

**Figure 17:**
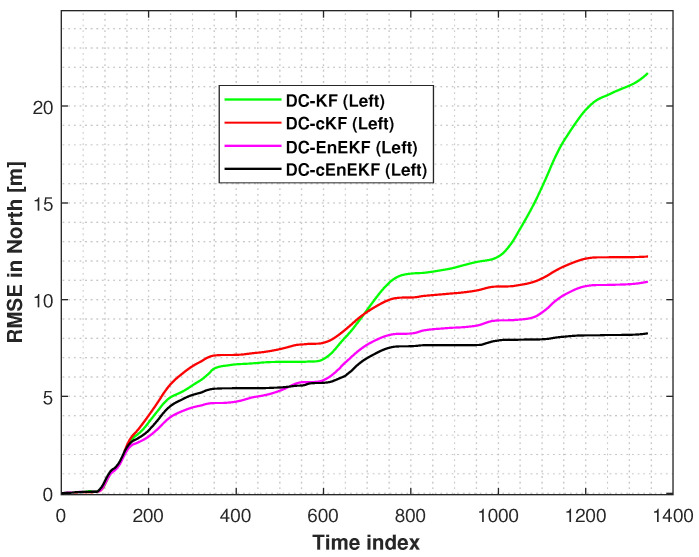
Left-foot northward position RMSEs obtained with DC–KF, DC–cKF, DC–EnEKF, and DC–cEnEKF on Path 3.

**Figure 18:**
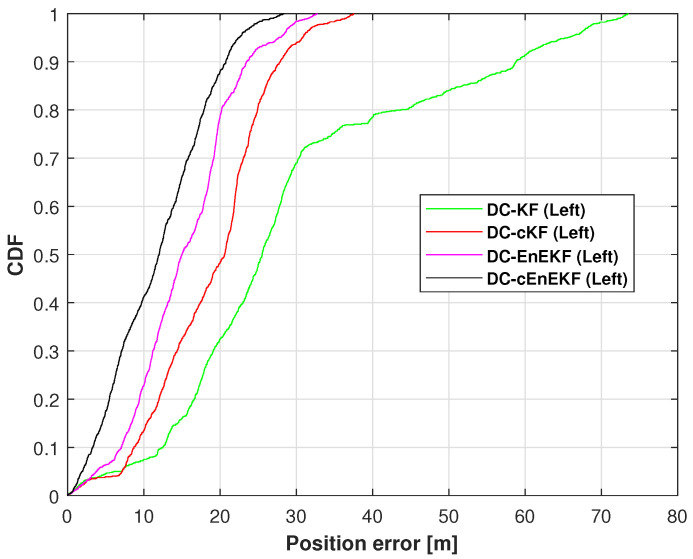
Left-foot position error CDFs derived with DC–KF, DC–cKF, DC–EnEKF, and DC–cEnEKF on Path 3.

Figure 16 and Figure 17 show the left-foot eastward and northward position RMSEs obtained with DC–KF, DC–cKF, DC–EnEKF, and DC–cEnEKF on Path 3. From the figures, it can be seen that all the solutions have accumulated errors. Noted that we consider the INS-based navigation in this work, although we propose the improved method to reduce the accumulated error, we can not eliminate this problem. If the pedestrian walking distance is too long, the method proposed in this paper will also have error accumulation, but we want to reduce the trend of error accumulation. From Figure 16 and Figure 17, we can see that the proposed method has the smallest accumulated errors. Left-foot position RMSEs obtained with DC–KF, DC–cKF, DC–EnEKF, and DC–cEnEKF on Path 3 are listed in Table 5. From this table, we can see that our proposed method can improve the localization accuracy by about 33.24% and 19.35% compared with the DC-cKF and DC-EnEKF. Left-foot position error CDFs derived with DC–KF, DC–cKF, DC–EnEKF, and DC–cEnEKF on Path 3 are shown in Figure 18. We can also see that the proposed method has the smallest position error at 0.9, which also shows that the proposed method is effective in reducing the localization error.

## 5. Conclusions

To improve the information accuracy of the IMU-based pedestrian localization, this study proposed a novel DC adaptive EnEKF. A pedestrian localization framework was first designed that employed two foot-mounted IMUs, with one unit securely attached to each foot. Triaxial acceleration, angular rate, and magnetic field measurements were simultaneously captured by both sensors. The raw data from each foot were subsequently processed by an independent data fusion filter to estimate the corresponding foot position. Next, a new data fusion model was derived, in which the quaternions used in the inertial navigation system (INS) computation were incorporated into the traditional INS error vector. Based on this model, the DC-based EnEKF was designed. In this method, the EnEKF was first employed to use ensemble statistics to address the nonlinear and non-Gaussian problems in a data fusion model. Subsequently, it was modified into the cEnEKF to mitigate the effects of CMN. Additionally, the DC was further incorporated to impose tighter restrictions on the left-foot and right-foot positions estimated by the EnEKF algorithm, thereby enhancing the overall solution consistency. Experimental results from two distinct practical scenarios confirm the efficacy and superior performance of the proposed framework. Future research will investigate how the proposed framework can be extended to better handle multi-rate sampling processes.

## Figures and Tables

**Table 1:** Technical specifications of the employed IMUs.

Parameter	Value
Number of axes	3
Dimensions	36.30×30.35×10.80 mm
Heading accuracy (dynamic)	1.0°|2.0°1σ RMS
Heading accuracy (static)	0.5°|1.0°1σ RMS
Operating temperature	0 °C ∼ 50 °C

**Table 2:** Main specifications of the RTK receiver (*D* means baseline length, same model as in [26]).

Parameter	Value
Horizontal positioning accuracy	$\pm \left(8+1\times 10^{6}\times D\right)\mathrm{mm}$
Vertical positioning accuracy	$\pm \left(15+1\times 10^{6}\times D\right)\mathrm{mm}$
Physical dimensions	119mm×119mm×85 mm
Mass	0.73 kg
Operating temperature range	−45 °C ∼ +75 °C

**Table 3:** Left-foot position RMSEs obtained with DC–KF, DC–cKF, DC–EnEKF, and DC–cEnEKF on Path 1.

Method	East (m)	North (m)	Mean (m)
INS	49.39	3.10	26.25
DC-KF	20.25	5.32	12.79
DC-cKF	7.34	2.72	5.03
DC-EnEKF	9.48	4.49	6.99
DC-cEnEKF	4.76	2.52	3.64

**Table 4:** Left-foot position RMSEs obtained with DC–KF, DC–cKF, DC–EnEKF, and DC–cEnEKF on Path 2.

Method	East (m)	North (m)	Mean (m)
INS	10.58	20.51	15.55
DC-KF	12.55	17.53	15.04
DC-cKF	7.93	3.37	5.51
DC-EnEKF	5.79	3.08	4.44
DC-cEnEKF	3.85	2.13	2.99

**Table 5:** Left-foot position RMSEs obtained with DC–KF, DC–cKF, DC–EnEKF, and DC–cEnEKF on Path 3.

Method	East (m)	North (m)	Mean (m)
INS	56.67	31.50	44.09
DC-KF	25.93	21.70	47.63
DC-cKF	16.23	12.23	14.23
DC-EnEKF	12.63	10.93	11.78
DC-cEnEKF	10.73	8.26	9.50

## Data Availability

The original contributions presented in the study are included in the article; further inquiries can be directed to the corresponding author.

## Abbreviations

The manuscript employs the following abbreviations:

BDS	BeiDou navigation satellite system
CDF	Cumulative distribution function
CMN	Colored measurement noise
EKF	Extended Kalman filter
EnEKF	Ensemble extended Kalman filter
GPS	Global positioning system
GNSS	Global navigation satellite system
IMU	Inertial measurement unit
INS	Inertial navigation system
KF	Kalman filter
LiDAR	Light detection and ranging
LOS	Line-of-sight
LSTM	Long short-term memory
DC	Distance constraint
NLOS	Non-line-of-sight
PPP	Precise pointing positioning
RFID	Radiofrequency identification
R-T-S	Rauch-Tung-Striebel
RMSE	Root mean square error
UAV	Uncrewed aerial vehicles
UWB	Ultrawide Band
ZUPT	Zero velocity update
